# Supplementary material for: A network embedding approach to identify active modules in biological interaction networks
Source: Life Sci Alliance. 2023 Jun 20;6(9):e202201550. doi: 10.26508/lsa.202201550 (PMC10282331; doi:10.26508/lsa.202201550)
Supplement: Supplementary file 7 [file LSA-2022-01550_TableS7.docx]

| **Metric** | **Artificial dense network** | **Real network** |
| --- | --- | --- |
| Number of vertices | 5980 | 5980 |
| Alpha coefficient | 1.908 | 1.602 |
| R square | 0.876 | 0.924 |
| Mean of neighbor connectivity | 42.207 | 47.720 |

**Supplemental Table S2. Comparison of artificial and real biological network.** Comparison of several metrics associated, on the one hand, an artificial dense network generated with the extended Barabasi-Albert model using 3 initial nodes and setting parameters p and q to 0.09 and 0.7 respectively and, on the other hand, a subnetwork containing the same number of vertices extracted from the STRING database.
